# Evaluating the Performance of Peak Calling Algorithms Available for Intracellular G-Quadruplex Sequencing

**DOI:** 10.3390/ijms26031268

**Published:** 2025-01-31

**Authors:** Yuqi Wang, Ke Xiao, Tiantong Tao, Rongxin Zhang, Huiling Shu, Xiao Sun

**Affiliations:** State Key Laboratory of Digital Medical Engineering, School of Biological Science and Medical Engineering, Southeast University, Nanjing 211189, China; wangyq0304@163.com (Y.W.); kexiao@seu.edu.cn (K.X.); taott2k@seu.edu.cn (T.T.); rongxinzhang@outlook.com (R.Z.); huilingshu@seu.edu.cn (H.S.)

**Keywords:** G-quadruplex (G4), peak calling algorithms, performance comparison

## Abstract

DNA G-quadruplexes (G4) are non-canonical DNA structures that play key roles in various biological processes. Antibody-dependent sequencing is an important tool for identifying intracellularly formed DNA G4s, and peak calling is a crucial step in processing the sequencing data. As the applicability of existing peak calling algorithms to intracellular G4 data has not been previously assessed, we systematically compared and evaluated these algorithms to determine those best suited for G4 detection. We selected seven representative candidates from 43 published peak calling algorithms for detailed evaluation. The performance of each candidate on six published intracellular G4 sequencing datasets (GSE107690, GSE145090, GSE133379, GSE178668ChIP-seq, GSE178668CUT&Tag, GSE221437) were assessed by precision and recall against customized benchmarks integrating results from multiple algorithms, as well as consistency with known G4 information (pG4 predicted by pqsfinder, oG4 from GSE63874, and multi-cell-line conserved G4s) and epigenetic signals. We identified MACS2, PeakRanger, and GoPeaks as the most effective algorithms for analyzing intracellular G4 sequencing data, and attributed their superior performance partially to the distribution model of sequencing reads/fragments used in the hypothesis testing step of the peak calling procedures. These findings provide guidance and rationale for selecting peak callers appropriate for intracellular G4 data.

## 1. Introduction

A G-quadruplex (G4) is a non-canonical secondary structure of DNA or RNA formed by a guanine-rich sequence. In a G4, four guanine bases form a plane called a G-tetrad through Hoogsteen hydrogen bonding, with multiple G-tetrads stacking on each other [1]. Both DNA and RNA G4s play crucial roles in various biological processes within cells [2,3]. Notably, G4s formed by genomic DNA are considered vital to gene expression regulation, DNA replication, maintenance of genome stability, and telomere maintenance [4,5,6]. Moreover, they are implicated in cancer and other diseases, highlighting their potential as therapeutic targets [7].

To understand the mechanisms underlying the functions of these DNA G4s, direct high-resolution capture of their formation within an intracellular chromatin context is essential [8]. Several methods, combining high-throughput sequencing with G4-specific antibodies, have been proposed to yield genome-wide maps detailing on the positions and frequencies of G4 formation in living cells [9]. The first protocol for mapping G4s intracellular, known as G4 ChIP-seq, was developed by Hänsel-Hertsch et al., using an engineered antibody called BG4 [10]. Similarly, Zheng et al. proposed a protocol using an artificial G4 probe protein, G4P, to capture G4s in living cells through a ChIP-Seq-based approach [11]. Moreover, Lyu et al. combined BG4 with the Tn5-based CUT&Tag procedure, establishing the first G4 CUT&Tag method for genome-wide profiling of native G4s [12]. Collectively, these methods produce high-throughput sequencing reads across the genome, providing insights into intracellular G4s formed.

Peak calling is a crucial step in analyzing intracellular G4 sequencing data, as it identifies the regions of interest, i.e., intracellular G4 sites, by analyzing stacks of aligned reads across the genome. The peak calling process can be divided into two main steps: first, identifying regions enriched with DNA reads/fragments as candidate peaks across the entire genome, and second, subjecting these candidates to hypothesis testing to determine their statistical significance [13]. Based on significance thresholds, candidate peaks are then ranked and filtered accordingly. However, peak calling results can be affected by factors such as experimental noise and the accessibility of target regions, which are commonly observed across datasets. To mitigate these challenges, different algorithms employ distinct strategies to optimize the two main steps of peak calling: identifying enriched regions and testing their statistical significance [14].

Several tools are available for intracellular G4 peak calling. Most published experiments [10,11,12], regardless of the antibody used (BG4 or G4P) or sequencing technique employed (ChIP-Seq or CUT&Tag), have utilized MACS2 [15] for peak calling. Exceptions include the studies by Hui et al. [16], which employed SEACR for analyzing G4-CUT&Tag data, and Lago et al. [17], which applied HOMER to their G4 ChIP-Seq data. The number of peaks generated by these three algorithms is comparable, and the validity of the peaks is supported by the presence of typical G4 motifs (G_x_N_1-7_G_x_N_1-7_G_x_N_1-7_G_x_N_1-7_, where x ≥ 3 and N can be any base), genomic annotations, or chromatin accessibility. Notably, there are over 40 peak calling algorithms available, but only a limited number have been used in G4-related studies. Although the performance of some of these algorithms have been compared and evaluated [13,18,19], their applicability to intracellular G4 sequencing data have yet to be thoroughly assessed. This is important because G-quadruplex (G4) formation depends on specific sequence motifs and is usually narrower than histone modification or transcription factor binding sites. Therefore, systematic evaluation of peak calling algorithms is essential for accurate identification of G4 structures and better understanding of their biological functions and regulatory roles.

One significant challenge for the comparison of these methods is the absence of a “gold standard” dataset for benchmarking. For intracellular G4 sequencing results, there is a lack of methods that are as credible as the Sanger sequencing is for next-generation sequencing data. Even when studying the same cell type, different antibodies (BG4 or G4P) and sequencing methods (ChIP-Seq or CUT&Tag) yield overlapping yet distinct sets of peaks [11,12,20], highlighting the difficulty and complexity of establishing a standardized test.

In this study, we focused on DNA G4s and the peak calling methods that might be suitable for the intracellular G4 sequencing data. We selected seven algorithms—MACS2 [15], HOMER [21], GEM [22], SICER [23], PeakRanger [24], GoPeaks [25], and SEACR [26]—based on criteria including original publications, documentation availability, usability, maintenance status, and underlying design principles, and evaluated the operating characteristics of them on intracellular G4 sequencing data. We prepared benchmark datasets using an integration strategy for benchmarking purposes. The validity of the identified G4 peaks was assessed based on the distributions of putative G4 motifs and associated epigenetic modifications. Additionally, we compared the effects of different candidate peak identification strategies and different hypothesis testing models on G4 data analysis. By synthesizing results across these analyses, we identified the most suitable peak calling algorithms for intracellular G4 sequencing to enhance their performance.

## 2. Results and Discussion

### 2.1. Evaluating the G4 Benchmark Dataset

To assess the reliability of the G4 benchmark datasets established by integrating results from multiple algorithms (as described in Section 3), we calculated the distance of sequencing signals between replicate samples at each benchmark peak. The distance value (Formula 1) measures the similarity of the sequencing fragment distribution based on the correlation coefficient of the signal vectors from the two replicates within a 1000-bp window. Smaller distance values indicate greater reproducibility of G4 formations across replicates, reflecting event stability. Five of the six test datasets, containing replicate data, were included in this evaluation.

**Figure 1:**
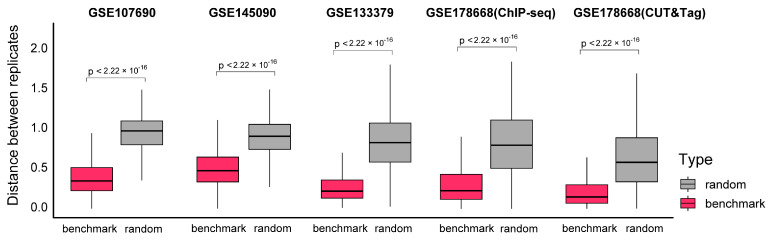
Evaluation of G4 benchmark datasets by signal distance between replicates. The x-axis represents different datasets and groups, and the y-axis represents distances between the signal vectors from two technical replicates within the same windows. Different groups, the benchmark and random, are indicated by different colors. Of the six test datasets utilized in this study, five containing technical replicates were selected for analysis, while GSE221437 was excluded from this evaluation due to the absence of replicate samples. Hypothesis testing between different groups was performed using the Wilcoxon test, and 2.22 × 10^−16^ is the default lower limit printed by the R package.

The analysis indicated that the mean distance values for the five G4 benchmark datasets ranged between 0.2 and 0.5, with two datasets showing mean values below 0.25 (Figure 1). Compared to randomly selected windows, the benchmark datasets exhibit significantly lower distance values, with *p*-values less than 2.22 × 10^−16^, which is the default lower limit printed by the R package. This consistent pattern of low distance values highlights the stability of G4 benchmark datasets and supports their reliability. These findings confirm that the benchmark datasets provide a robust reference for evaluating the performance of candidate algorithms in identifying G4 peaks.

### 2.2. Overall Performance of the Candidate Algorithms

Since the GoPeaks and SEACR algorithms do not provide *p*-values for identified G4 peaks, this analysis focuses solely on the results from MACS2, HOMER, GEM, SICER, and PeakRanger. We ranked all identified G4 peaks in ascending order based on their *p*-values, then applied thresholds to select the top 10², 10³, 10⁴, 10⁵, and 10⁶ G4 peaks, corresponding to different confidence levels. For each group, the harmonic mean (HM) score, equally weighting precision and recall, was calculated to assess algorithm performance. Higher scores indicate better suitability for analyzing intracellular G4 sequencing data.

**Figure 2:**
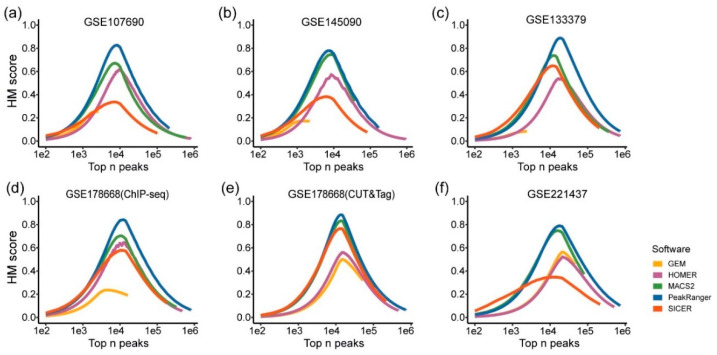
Comparison of HM scores for G4 peak identified by candidate algorithms. (**a**–**f**) correspond to the results of the six datasets, respectively. The x-axis represents different numbers of G4 peaks, with higher values indicating a greater number of peaks. The y-axis represents the HM score, ranging from 0 to 1.0. Algorithms are indicated by different colors.

The HM scores for the candidate algorithms across different test datasets are summarized in Figure 2. The results demonstrate that for all the six benchmark datasets, as the threshold increases, the HM scores for G4 peak data initially rise, peak around the value of 10⁴, and then decline (Figure 2a–f). These peaks at the 10⁴ threshold occur because the six benchmark datasets used as references contain approximately 10⁴ G4 peaks, consistent with the findings of Robert et al. [8]. Additionally, a lower threshold, indicating fewer G4 peaks being selected, decreases recall, whereas a higher threshold reduces precision, both resulting in a decline of the HM score.

Among the algorithms, PeakRanger and MACS2 demonstrate superior performance. The maximum HM scores for PeakRanger range from 0.78 to 0.89 (blue curve in Figure 2a–f), and those for MACS2 range from 0.67 to 0.84 (green curve in Figure 2a–f). Both algorithms exhibit higher scores than the others, indicating that PeakRanger and MACS2 outperform the remaining algorithms in combined precision and recall. Notably, GEM identified only 2000 G4 peaks in three test datasets (Figure 2a–c), which is significantly lower than the counts produced by other algorithms, resulting in a shorter curve length and a narrower score range. Overall, these findings highlight that PeakRanger and MACS2 demonstrate superior precision and recall in G4 peak identification, making them well-suited for peak identification in intracellular G4 sequencing data.

### 2.3. Evaluating Candidate Algorithms Based on Known G4 Information

The results of individual peak calling processes could be verified with G4 information available from other sources, e.g., putative G4s (pG4s), observed G4s (oG4s), and active G4s (aG4s) (Supplementary Figure S1). In particular, pG4s, also known as G4 motifs, refer to predicted G4 sites with DNA sequences that may potentially form G4 structures [27] and oG4s are identified by in vitro sequencing techniques, such as G4-seq [28], and are often regarded as sites where G4 structures can form in the in vitro environment. Lastly, aG4s are the sites that have been identified and validated in multiple cell lines through intracellular G4 sequencing methods, thus they are considered regions with intracellular G4s [29]. An identified peak is more likely to be caused by a bona fide intracellular G4 structure if it is overlapping with any pG4, oG4 or aG4 site, and thus the latter can serve as supporting evidence for the authenticity of the former.

Therefore, we calculated the proportions of peaks overlapping with pG4s, oG4s, and aG4s for each of the candidate peak calling algorithm (Table 1). Overall, the results generated by SEACR show the highest proportions of pG4-, oG4-, and aG4-overlapping peaks, followed by those generated by SICER, MACS2, PeakRanger, and GoPeaks, while the results from HOMER and GEM exhibit the lowest proportions. This trend can be consistently observed across all six test datasets.

**Figure 3:**
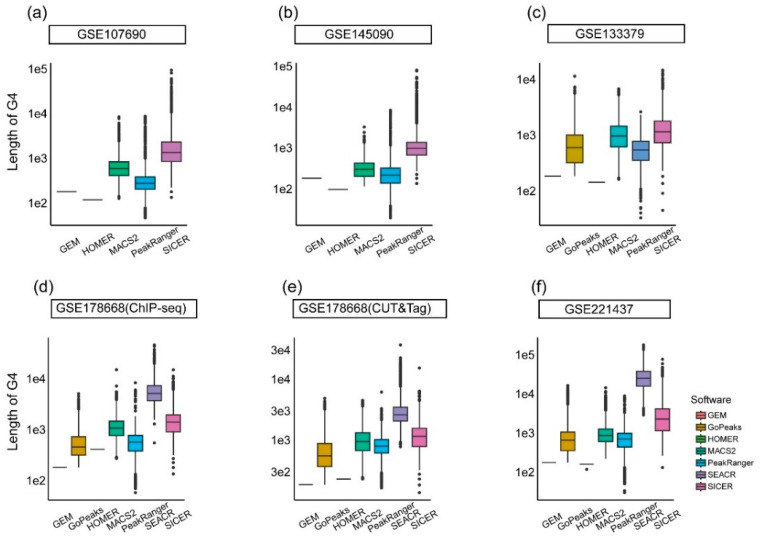
Width distribution of G4 peaks identified by the candidate algorithms. The x-axis represents different algorithms, and the y-axis indicates the width of G4 peaks identified by each algorithm. (**a**,**b**) use test datasets with single-end sequencing data, applicable to only five of the candidate algorithms. (**c**) uses a test dataset compatible with six algorithms, while (**d**–**f**) utilize test datasets that accommodate all seven algorithms.

Logically, wider peaks will cover longer DNA regions, and thus have a higher probability of overlapping with pG4, oG4, or aG4 sites. The average widths of peaks identified by SEACR and SICER, across different test datasets, range from 3000 bp to 30,000 bp and from 1330 bp to 3870 bp, respectively (Figure 3). The low resolution of both algorithms partially accounts for the high proportions of overlapping peaks generated by them. In contrast, HOMER and GEM harvest peaks significantly narrower than those identified by other methods, with fixed values of 180 bp and 201 bp, respectively. This explains the low proportions of overlapping peaks produced by them. All four algorithms either generate wider peaks, which correspond to lower resolutions, or fail to produce a sufficient number of peaks that can be validated with G4 information available from other sources. This suggests that these algorithms may not be optimal choices for the peak calling processes of intracellular G4 sequencing data.

The remaining three candidates, i.e., MACS2, PeakRanger, and GoPeaks, all yield G4 peaks with average widths of less than 1000 bp, which is consistent with the findings reported by Robert et al. [8] and Li et al. [20]. Taking into consideration the appropriate peak widths and the moderately high proportion of overlapping peaks, the G4 peaks identified by MACS2, PeakRanger, and GoPeaks are more likely to represent authentic intracellular G4s.

### 2.4. Evaluating the Validity of Algorithm-Specific G4 Peaks

Since the sets of G4 peaks identified by different algorithms partially overlap, we define these overlapping regions shared by all the candidates as “common G4 peaks”. After removing the common G4 peaks from the results of each algorithm, the remaining ones are referred to as “algorithm-specific G4 peaks”. The common G4 peaks are intuitively reliable, as they have been validated by all the candidate algorithms. However, it is still necessary to evaluate the algorithm-specific G4 peaks for each candidate, as their validity specifically reflects the differences in performance of the candidates. Due to the previously mentioned low precision and recall based on the benchmark, as well as the inappropriate peak width and quantity, we excluded SICER and SEACR from further comparisons. The subsequent analyses focused exclusively on MACS2, HOMER, GEM, PeakRanger, and GoPeaks.

**Figure 4:**
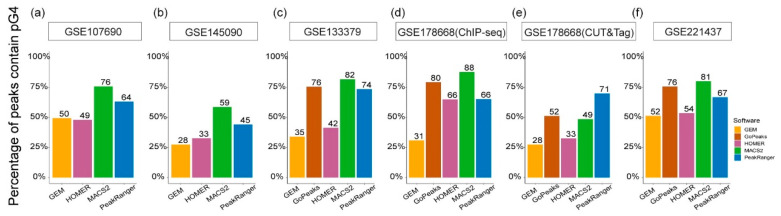
Proportion of pG4 sites in algorithm-specific G4 peaks from different test datasets. The x-axis represents different algorithms, and the y-axis indicates the percentage of overlapping pG4 sites. (**a**,**b**) use single-end test datasets, applicable to four algorithms to be run, while (**c**–**f**) employ test datasets that accommodate all five algorithms.

Again, pG4 sites, i.e., the G4 motifs, were utilized to validate the reliability of the G4 peaks identified by the candidate algorithms (Figure 4). In four out of the six test datasets, over 70% of the MACS2-specific G4 peaks overlap with pG4 sites, and in only one test dataset, the proportion of the pG4-overlapping peaks is less than half (approximately 49%). Similarly, PeakRanger-specific G4 peaks exhibit a trend of over 60% overlap with pG4 sites in five of the six test datasets, and more than 70% of the GoPeaks-specific peaks overlap with pG4s in three of the four datasets applicable to the algorithm. In contrast, the proportions of pG4-overlapping peaks in the HOMER- and GEM-specific results are below 50% in most test datasets.

G4s exhibit a strong tendency to localize in promoters and regions characterized by high chromatin accessibility, as they are closely associated with biological processes such as transcription [17]. Consequently, epigenetic marks that indicate chromatin state and transcription activity can also be employed as supporting materials for validating the authenticity of these algorithm-specific peaks. We employed ATAC-seq signals to assess chromatin accessibility, alongside four types of histone modifications: H3K4me1, which is usually associated with enhancers; H3K4me2 and H3K4me3, which may regulate RNA polymerase II pausing; and H3K27ac, which is generally considered a marker of active enhancers and promoters [30].

**Figure 5:**
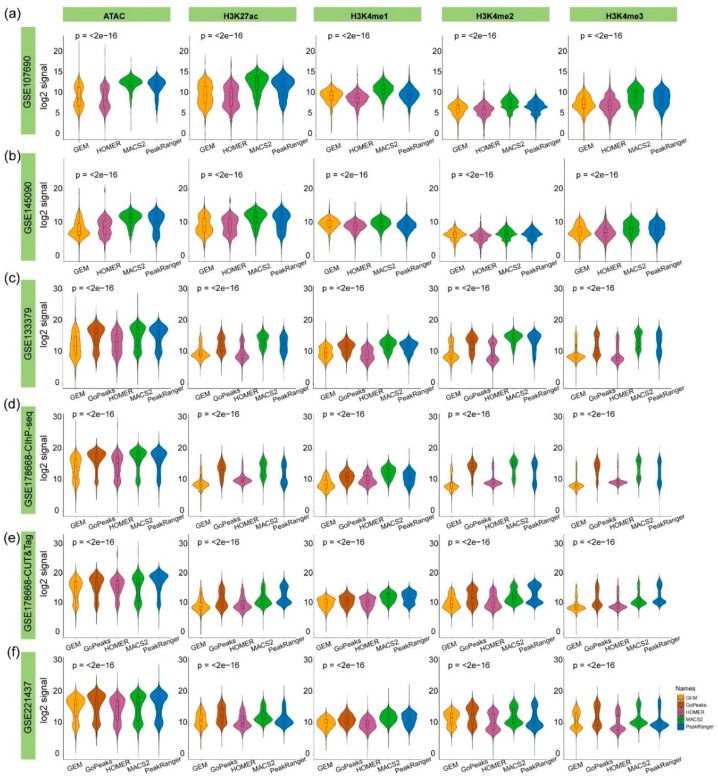
Comparison of epigenetic signals at algorithm-specific G4 peaks across different test datasets. The x-axis represents different algorithms, and the y-axis represents the log-transformed signal values of chromatin accessibility (ATAC) or histone modifications (H3K27ac, H3K4me1, H3K4me2, H3K4me3). (**a**,**b**) are derived from single-end test datasets applicable to four algorithms, (**c**–**f**) are based on test datasets applicable to all five algorithms. The *p*-values displayed were calculated using the Kruskal–Wallis test to assess the statistical significance of differences among the groups identified by different algorithms.

The strength values of these five types of epigenetic signals were calculated at the algorithm-specific G4 peaks. The MACS2-, PeakRanger-, and GoPeaks-specific peaks exhibit significantly higher chromatin accessibility and histone modification signals compared to those from HOMER and GEM (Figure 5). This indicates stronger consistency of these algorithm-specific G4 peaks with known biological characteristics of intracellular G4 functions. Consequently, the algorithm-specific G4 peaks identified by MACS2, PeakRanger, and GoPeaks are more likely to be involved in active biological processes, reinforcing their authenticity.

### 2.5. Overall Evaluation of the Candidate Algorithms

According to the performance evaluated using the benchmark datasets, along with validation against known pG4, oG4, and aG4 sites, as well as the validity of the algorithm-specific peaks, we recommend three methods that excel across all the factors as tools for intracellular G4 peak calling: MACS2, PeakRanger, and GoPeaks (Table 2). In contrast, the other candidates either performed poorly on the benchmark, or had resolution unsuitable for G4 data, or generated peaks not consistent with those known G4 information or epigenetic signals, making them less suitable for the peak calling task for intracellular G4 sequencing data.

Furthermore, we aimed to investigate the characteristics that might influence the performance of these algorithms on intracellular G4 sequencing data. The two stages of peak calling—identifying enriched regions as candidate peaks and evaluating their statistical significance against controls through hypothesis testing—were assessed separately. Since SEACR and GEM do not conform to this framework due to their distinct methodologies [26,31], our analysis was restricted to MACS2, HOMER, SICER, PeakRanger, and GoPeaks. We compared the strategies employed by these algorithms at each step to determine their impact on G4 peak identification.

In the step of candidate peak identification, there are at least two available strategies. MACS2, HOMER, and PeakRanger employ a sliding window approach to scan the genome for candidate peaks, while SICER and GoPeaks segment the genome into fixed intervals. Across all test datasets, over 96% of intracellular G4 sites were successfully identified by all five algorithms (Supplementary Figure S2), indicating that both the sliding window and fixed-interval strategies are effective for capturing intracellular G4 sites with no discernible difference in the results between the two strategies.

**Figure 6:**
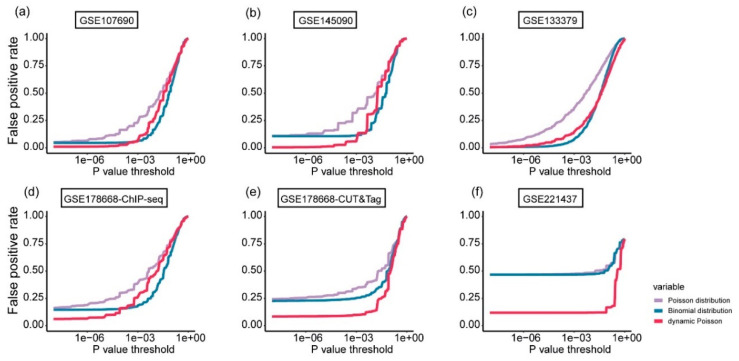
False positive rate comparison of the hypothesis testing using the three distribution models—Poisson, binomial, and dynamic Poisson—across six test datasets. The x-axis represents the *p*-value, while the y-axis shows the corresponding false positive rate. (**a**–**f**) correspond to the results of the six datasets, respectively. Models are indicated by different colors.

In the second step, i.e., evaluating the significance of the candidate peaks, three distribution models for the sequencing reads/fragments are available: the Poisson model employed by SICER and HOMER, the dynamic Poisson model used by MACS2, and the binomial model utilized by GoPeaks and PeakRanger. We assessed the impact of these distribution models on G4 peak identification by analyzing the false positive rates varying with *p*-values of the hypothesis testing, across the six test datasets (Figure 6).

The dynamic Poisson model consistently achieves the lowest false positive rates, especially with significance thresholds $\leq 10^{-3}$. This model outperforms both the Binomial and Poisson models and demonstrates its effectiveness in G4 peak identification. For most test datasets, when looser thresholds (*p*-value $\geq 10^{-3}$) are applied, the binomial model outperforms the other two models. In certain cases, such as the GSE133379 dataset, it may perform comparably to the dynamic Poisson model at thresholds $\leq 10^{-3}$, likely due to a higher sequencing depth and a more uniform fragment distribution that favor the binomial assumption.

Furthermore, the Poisson model consistently produces the highest false positive rates across all test datasets and thresholds, highlighting its limitations in accurately identifying G4 peaks. These findings provide a plausible explanation of the superior performance of algorithms such as MACS2 (using the dynamic Poisson model) and GoPeaks and PeakRanger (utilizing the binomial model) compared to SICER and HOMER (relying solely on the Poisson model) in G4 identification.

## 3. Methods

### 3.1. Selection of G4 Sequencing Datasets

Six G4 sequencing datasets, referred to as ‘test datasets’ from the Gene Expression Omnibus (GEO) were selected to evaluate the performance of the candidate algorithms. These test datasets included two derived from the K562 cell line and four from the HEK293T cell line. Detailed characteristics of each test dataset are summarized in Table 3. The selected test datasets encompass two distinct experimental approaches—ChIP-seq and CUT&Tag—utilizing two different antibodies, BG4 and G4P, and incorporating both paired-end and single-end library types.

### 3.2. Selection of Peak Calling Algorithms

Forty-three peak calling algorithms were reviewed (Supplementary Table S1). Based on criteria including original publications, availability of documentation, usability, maintenance status, and underlying design principles, seven algorithms were selected for detailed evaluation with G4 sequencing data: MACS2, HOMER, GEM, SICER, PeakRanger, GoPeaks, and SEACR. The algorithms differ in their strategies for identifying candidate peaks and performing hypothesis testing. MACS2, HOMER, and PeakRanger utilize a sliding window approach for peak identification, while SICER and GoPeaks divide the genome into fixed intervals. For hypothesis testing, MACS2 applies a dynamic Poisson model for the distribution of sequencing reads/fragments, SICER and HOMER use Poisson models, and GoPeaks and PeakRanger employ binomial models. SEACR, which adjusts thresholds using a global background signal distribution, and GEM, which integrates binding event discovery with motif identification via probabilistic modeling, do not conform to this classification framework.

### 3.3. G4 Sequencing Data Analysis Pipeline

Raw sequencing data were quality-checked and adapter-trimmed using Trim Galore (v0.6.6) [32]. Reads were aligned to the hg19 reference genome with Bowtie2 (v2.3.5.1) [33], and alignment results were stored in BAM format using Samtools (v1.9) [34]. Duplicate reads were removed with Picard (v2.23.4) MarkDuplicates, and genomic blacklist regions were excluded using Bedtools (v2.29.1) intersect [35].

### 3.4. G4 Peak Identification and Preprocessing

To evaluate algorithm performance, peak callers were applied to six test datasets under the lowest significance thresholds to generate unfiltered candidate peaks. Specific configurations with the lowest thresholds were used for each algorithm: GEM (q=0, fold=1), MACS2 (q=1, f=BAMPE for paired-end data), HOMER (F=1, P=1, L=1, LP=1, C=0, fdr=1), SICER (fdr=1), PeakRanger (q=1, p=0.999), and SEACR (relaxed mode). For GoPeaks, *p*-values were adjusted in the code to vary peak counts, as the algorithm does not provide *p*-values for each interval. Performance differences across test datasets were observed due to algorithm-specific limitations, such as GoPeaks supporting only paired-end data and SEACR requiring paired-end data with an IgG antibody control (Supplementary Table S2). Candidate peaks identified by each algorithm were ranked by *p*-value or FDR. The top 10,000 high-confidence intervals were selected as G4 peaks [20].

### 3.5. Construction of G4 Benchmark Datasets

The benchmark was generated by identifying the overlap of G4 peaks detected by multiple algorithms within each test dataset (Supplementary Figure S3). For each algorithm, the peaks were ranked by their *p*-values or corresponding significance scores, and the top 10,000 peaks were regarded as identified peaks for that algorithm. Peaks identified by more than half of the algorithms were included in the benchmark. The remaining ones from each algorithm were taken as algorithm-specific peaks.

For the test datasets containing multiple replicate samples, the first replicates from each test dataset were employed for construction of the benchmark, and the other replicates were used for validation of the benchmark.

**Figure 7:**
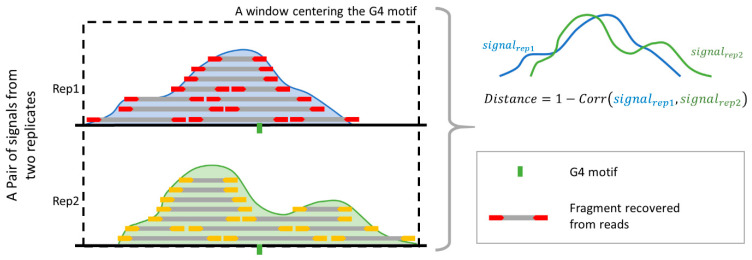
Procedure for validating the reproducibility of benchmark peaks across replicates. The benchmark peak profile (blue curve) is identified from the first replicate and associated with the nearest G4 motif. The second replicate is used for validation, with its signal profile (green curve) extracted within the same 1000 bp window centered on the G4 motif. The “distance” metric, defined as 1 minus the Pearson’s correlation coefficient between the signal vectors, quantifies the consistency between replicates, with smaller values indicating higher reproducibility.

To validate the benchmark, a “distance” value was introduced to evaluate the consistency of G4 signals across replicates (Figure 7). Here, the peaks from the benchmark were attributed to the G4 motifs nearest to the peak center. The signal values with a 1000-bp window centering these motifs, from both replicates, were saved as vectors for calculating the distance values. The distance is defined as:

(1) $$Distance=1-Correlation\left(signal_{rep1},signal_{rep2}\right)$$

where *signal_rep1_* and *signal_rep2_* represent the vectors generated within the same window from two technical replicates, and Correlation refers to the Pearson’s correlation coefficient between these two vectors. The distance value ranges from 0 to 2, where 0 indicates perfect correlation and 2 indicates no correlation. Smaller distance values reflect stronger signal consistency across replicates, suggesting that the intracellular G4 is stable and reliably present.

Additionally, random groups for each test dataset were generated by utilizing signal vectors from 10,000 randomly selected windows, serving as controls.

### 3.6. Calculating Precision, Recall, and Harmonic Mean for Each Candidate Algorithm

**Figure 8:**
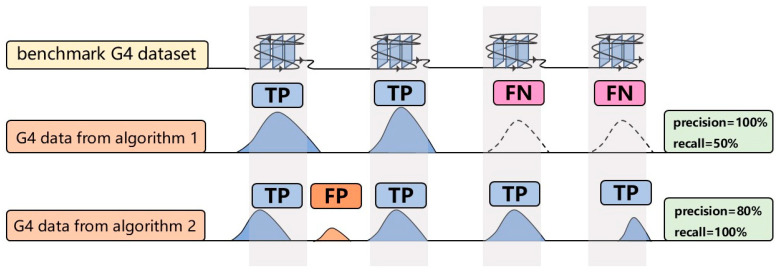
Precision and recall illustration. The first row shows the G4 benchmark dataset as the reference. The second and third rows depict G4 peaks identified by different algorithms. Blue peaks indicate true positives, orange peaks represent false positives, and dashed peaks denote false negatives.

Precision and recall of G4 peaks identified by each algorithm were calculated using the G4 benchmark dataset as the reference standard. The harmonic mean (HM score) of precision and recall was employed as the overall performance metric to emphasize balanced algorithm performance. The HM score, which increases only when both precision and recall are high, provides a balanced measure of algorithm performance. Precision is defined as the proportion of overlapping base pairs between algorithm-identified G4 peaks and the benchmark dataset, while recall represents the proportion of benchmark peaks overlapping with algorithm-identified peaks (Figure 8). The formulas are:

(2) $$Precision=\frac{TP}{\left(TP+FP\right)}$$

(3) $$Recall=\frac{TP}{\left(TP+FN\right)}$$

(4) $$HM\;score=\frac{2}{\left(\frac{1}{Precision}+\frac{1}{Recall}\right)}$$

TP (True Positive) represents algorithm-identified G4 peaks that overlap with the benchmark dataset. FP (False Positive) refers to algorithm-identified peaks that do not overlap with the benchmark dataset, while FN (False Negative) denotes benchmark dataset that do not overlap with algorithm-identified peaks.

### 3.7. Proportion of pG4, oG4, and aG4 in Candidate G4 Peaks

pG4 (putative G-quadruplexes) refers to potential G4 sites predicted from genomic DNA using computational tools. In this study, pG4 sites were predicted using pqsFinder [27]. oG4 (observed G-quadruplexes) refers to in vitro G4 sites identified through techniques like G4-seq [28], which stabilize G4s with PDS or K+. The oG4 data were obtained from GSE63874. aG4 (active G-quadruplexes), identified by Zhang et al. [29], represents G4 sites identified across multiple cancer cell lines, reflecting strong intracellular secondary structure formation. The proportions of G4 peaks identified by each algorithm overlapping with pG4, oG4, and aG4 were calculated using Bedtools intersect [35].

### 3.8. Chromatin Accessibility Data Processing

Chromatin accessibility and histone modification data for the K562 and HEK293T cell lines were obtained from GEO (K562: GSE215595, GSE176370, GSE127009, GSE127010, GSE213909; HEK293T: GSE178668). Chromatin signals over G4 peaks were calculated using bigWigAverageOverBed (v2) [36] and log-transformed. Signal differences across G4 peaks were analyzed using the Kruskal–Wallis test [37].

### 3.9. Construction of the G4 To-Be-Tested Datasets for Hypothesis Testing Evaluation

To evaluate hypothesis testing models, including Binomial, Poisson, and dynamic Poisson distributions, each G4 to-be-tested dataset was constructed with positive samples (known G4 sites) and negative samples (non-G4 sites). Sequencing fragment counts were calculated for each genomic position, and *p*-values were computed based on fragment density. A threshold was applied to identify G4 peaks, and false positive rates were calculated to assess model performance. The G4 benchmark dataset was used as the positive sample set for each test dataset.

Negative samples were generated by combining all unfiltered G4 peaks identified by candidate algorithms and excluding peaks overlapping the G4 benchmark dataset, those predicted by pqsFinder or G4Hunter [38], or these annotated by Tan et al. [11] (GSE133379). The remaining intervals were extracted from hg19 using Bedtools getfasta and filtered to remove sequences matching the pattern *G_3-5_N_1-7_G_3-5_N_1-7_G_3-5_N_1-7_G_3-5_*. A random selection of 10,000 intervals was used as the negative sample set. Positive and negative samples were then combined to construct the G4 to-be-tested datasets.

### 3.10. Reads/Fragment Distribution Models and False Positive Rate Evaluation

Three reads/fragments distribution models commonly used in hypothesis testing of peak calling algorithms—Poisson, Binomial, and dynamic Poisson distribution—were evaluated.

In the Poisson model, the number of sequencing fragments in the treatment replicate at a candidate G4 peak X follows a Poisson distribution:

(5) $$X\sim Pois\left(\lambda \right)$$

where the expected value λ is the fragment counts from the control replicate at the same site.

The dynamic Poisson model is similar to the Poisson model but with a dynamic λ, following the approach described by Zhang et al. [15]. For the control, genomic intervals of 1 kb, 5 kb, 10 kb, 50 kb, 100 kb, 500 kb, and 1000 kb are extended upstream and downstream of the candidate G4 peak. The average number of fragments are counted in each interval, as ($\lambda_{1k},\;\lambda_{5k},\;\lambda_{10k},\;\lambda_{50k},\;\lambda_{100k},\;\lambda_{500k},\;\lambda_{1000k}$), along with the genome-wide background ($\lambda_{all}$). The expected value of the fragment counts is set as $\lambda_{dynamic}=max\;\left(b,\;\lambda_{1k},\;\lambda_{5k},\;\lambda_{10k},\;\lambda_{50k},\;\lambda_{100k},\;\lambda_{500k},\;\lambda_{1000k},\;\lambda_{all}\right)$. The fragment counts in the treatment X follows the Poisson distribution:

(6) $$X\sim Pois\left(\lambda_{dynamics}\right)$$

In the binomial model, the fragment counts at the candidate G4 peak in the treatment follows a binomial distribution:

(7) $$X\sim B\left(n,p\right)$$

where n is the scaled number of sequencing fragments in the control, and p is estimated as the average fragment counts in all the candidate peak regions in the control divided by n.

The *p*-values were computed based on the random variable X, and the corresponding distribution. All calculations for the Poisson and Binomial models were performed using R (v4.1.0).

False positive rates were calculated as the proportion of G4 peaks identified at varying thresholds that did not overlap with known positive G4 peaks:

(8) $$False\;positive\;rate=\frac{FP}{\left(TP+FP\right)}$$

TP (True Positive) is the number of G4 peaks identified by the hypothesis testing models that overlap with the known positive G4 peaks dataset. FP (False Positive) is the number of identified G4 peaks that do not overlap with the positive G4 peaks dataset.

## 4. Conclusions

This study systematically evaluated seven peak calling algorithms—MACS2, HOMER, GEM, SICER, PeakRanger, GoPeaks, and SEACR—for intracellular G4 sequencing data. Benchmark datasets established by integrating results from multiple algorithms were generated, and their reliability was assessed based on peak similarity between replicate samples. Additionally, known G4 information, including pG4, oG4 and aG4, was employed to evaluate the results of these candidate algorithms. Furthermore, the performance of these algorithms was also assessed through the validity of the algorithm-specific peaks, by using pG4 sites and known epigenetic signals.

Among the seven candidate algorithms, SICER and SEACR generated high proportions of peaks overlapping with pG4s, oG4s, and aG4s, yet the width of these peaks was not optimal for G4 peak calling tasks. While HOMER and GEM could generate much narrower peaks, indicating higher resolution, they underperformed in other metrics. In contrast, MACS2, PeakRanger, and GoPeaks demonstrated superior performance. These three algorithms not only showed high precision and recall against benchmarks, but also produced a moderately high proportion of pG4-, oG4- and aG4-overlapping peaks with moderate widths. Moreover, they effectively identified biologically relevant G4 peaks enriched with known sequence motifs and other epigenetic signals. The successful application of these three algorithms to intracellular G4 data might be partially attributed to the distribution model of sequencing reads/fragments—either dynamic Poisson or binomial—employed during the hypothesis testing step of the peak calling procedures.

Given the diversity in sequencing methods, antibodies, and cell lines across the six published datasets we utilized, it is reasonable to conclude that the results in this study, i.e., the superior performance of MACS2, PeakRanger, and GoPeaks, are broadly applicable to most intracellular G4 sequencing data. Our findings provide practical guidance and rationale for selecting the peak caller in intracellular G4 identification, including the preparation of the evaluation process as well as the characteristics of the algorithms that could be considered. These results will promote the reliable detection of functional G4 structures across the genome.

Future efforts should focus on developing G4-specific peak calling algorithms that account for the unique sequence and functional characteristics of G4s, including their narrow peak width and sensitivity to chromatin context. Incorporating models tailored to G4 folding dynamics and motif specificity could improve the accuracy of G4 peak identification, thereby facilitating a better understanding of their roles in gene regulation and genomic stability. Such advancements would not only refine G4 mapping but also promote their use as biomarkers and therapeutic targets, thereby expanding the potential applications of G4 identification in molecular biology and clinical settings.

## Figures and Tables

**Table 1:** Proportion of pG4-, oG4-, and aG4-overlapping peaks generated by the candidate peak calling algorithms.

Candidate Algorithm	Proportion of pG4-Overlapping Peaks	Proportion of oG4-Overlapping Peaks	Proportion of aG4-Overlapping Peaks
SEACR	99–100%	79–100%	83–93%
SICER	76–98%	53–85%	44–83%
MACS2	76–95%	52–78%	54–83%
PeakRanger	66–91%	50–71%	60–82%
GoPeaks	73–91%	49–72%	58–88%
HOMER	50–82%	35–61%	54–78%
GEM	42–69%	29–52%	34–76%

**Table 2:** Summary of performance of peak calling algorithms.

Peak Calling Algorithm	Performance on Benchmark	Performance Based on Known G4 Information		Validity of Algorithm-Specific Peaks		Main Strategy
		Peak Width	Proportion of Overlapping Peaks	Percentage of pG4-Overlaping Peaks	Consistency with Epigenetic Signals	
MACS2	High HM score	Moderate	Moderately high	High	High	A sliding window approach + dynamic Poisson model
PeakRanger	Highest HM score	Moderate	Moderately high	High	High	A sliding window approach + binomial model
GoPeaks	-	Moderate	Moderately high	High	High	Segmenting genome into fixed intervals + binomial model
HOMER	Moderate HM score	Narrow	Low	Low	Low	A sliding window approach + Poisson model
GEM	Much fewer peaks	Narrow	Low	Low	Low	A distinct generative probabilistic model of ChIP data and genome sequence
SICER	Moderate HM score	Excessively high	High	-	-	Segmenting genome into fixed intervals + Poisson model
SEACR	-	Excessively high	High	-	-	Calling peaks from all non-zero signals based on the global distribution of background

**Table 3:** Summary of data characteristics for six test datasets.

GEO Accession Number	Cell Line	Antibody Type	Sequencing Technique	Library Type
GSE107690	K562	BG4	G4-ChIP-seq	Single-end
GSE145090	K562	BG4	G4-ChIP-seq	Single-end
GSE133379	HEK293T	G4P	G4P-ChIP-seq	Paired-end
GSE178668ChIP-seq	HEK293T	BG4	G4-ChIP-seq	Paired-end
GSE178668CUT&Tag	HEK293T	BG4	G4 CUT&Tag	Paired-end
GSE221437	HEK293T	BG4	G4 CUT&Tag	Paired-end

## Data Availability

The customized benchmark datasets that support the findings of this study are openly available in github at https://github.com/7YQXX3/G4-benchmark-datastes (accessed on 26 January 2025).

## Supplementary Materials

The following supporting information can be downloaded at: https://www.mdpi.com/article/10.3390/ijms26031268/s1.
