# Supplementary material for: Evaluating the Performance of Peak Calling Algorithms Available for Intracellular G-Quadruplex Sequencing
Source: Int J Mol Sci. 2025 Jan 31;26(3):1268. doi: 10.3390/ijms26031268 (PMC11818603; doi:10.3390/ijms26031268)
Supplement: Supplementary file 1 [file ijms-26-01268-s001.zip › ijms-3406546-supplementary.pdf]

Supplementary Files

Table S1. As of April 2022, a summary of published peak calling algorithms and their citation counts.

**Software**	**Article Title**	**Publication Year**	**Number of Citations**
**MACS2**	Model-based Analysis of ChIP-Seq (MACS)	2008	7325
**HOMER**	Simple combinations of lineage-determining transcription factors prime cis-regulatory elements required for macrophage and B cell identities	2010	5472
**SICER**	A clustering approach for identification of enriched domains from histone modification ChIP-Seq data	2009	658
**Hotspot**	Chromatin accessibility pre-determines glucocorticoid receptor binding patterns	2011	646
**spp-wtd**	Design and analysis of ChIP-seq experiments for DNA-binding proteins.	2008	575
**spp-mtc**	Design and analysis of ChIP-seq experiments for DNA-binding proteins.	2008	575
**spp-msp**	Design and analysis of ChIP-seq experiments for DNA-binding proteins.	2008	575
**CisGenome**	An integrated software system for analyzing ChIP-chip and ChIP-seq data	2008	560
**QuEST**	Genome-wide analysis of transcription factor binding sites based on ChIP-Seq data	2008	515
**SISSRs**	Genome-wide identification of in vivo protein-DNA binding sites from ChIP-Seq data	2008	425
**PeakSeq**	PeakSeq enables systematic scoring of ChIP-seq experiments relative to controls	2009	419
**F-seq**	F-Seq: a feature density estimator for high-throughput sequence tags	2008	254
**FindPeaks**	FindPeaks 3.1: a tool for identifying areas of enrichment from massively parallel short-read sequencing technology	2008	208
**GEM**	High Resolution Genome Wide Binding Event Finding and Motif Discovery Reveals Transcription Factor Spatial Binding Constraints	2012	161
**TM**	The role of chromatin accessibility in directing the widespread, overlapping patterns of Drosophila transcription factor binding	2011	142
**SEACR**	Peak calling by Sparse Enrichment Analysis for CUT&RUN chromatin profiling	2019	124
**ZINBA**	ZINBA integrates local covariates with DNA-seq data to identify broad and narrow regions of enrichment, even within amplified genomic regions	2011	115
**RSEG**	Identifying dispersed epigenomic domains from ChIP-Seq data	2011	110

**ChIPDiff**	An HMM approach to genome-wide identification of differential histone modification sites from ChIP-seq data	2008	107
**DFilter**	Uniform, optimal signal processing of mapped deep-sequencing data	2013	101
**CCAT**	A signal–noise model for significance analysis of ChIP-seq with negative control	2010	99
**BayesPeak**	BayesPeak: Bayesian analysis of ChIP-seq data	2009	95
**Sole-Search**	Sole-Search: an integrated analysis program for peak detection and functional annotation using ChIP-seq data	2010	92
**Hpeak**	HPeak: an HMM-based algorithm for defining read-enriched regions in ChIP-Seq data	2010	84
**MOSAiCS**	A Statistical Framework for the Analysis of ChIP-Seq Data	2011	70
**Qeseq**	Picking ChIP-seq peak detectors for analyzing chromatin modification experiments.	2012	52
**Gene Track**	GeneTrack - a genomic data processing and visualization framework	2008	46
**W-ChIPPeaks**	W-ChIPeaks: a comprehensive web application tool for processing ChIP-chip and ChIP-seq data	2011	40
**peakzilla**	Identification of transcription factor binding sites from ChIP-seq data at high resolution	2013	38
**MUSIC**	MUSIC: identification of enriched regions in ChIP-Seq experiments using a mappability-corrected multiscale signal processing framework.	2014	36
**TPIC**	Shape-based peak identification for ChIP-Seq	2011	35
**BCP(TF)**	Genome-Wide Localization of Protein-DNA Binding and Histone Modification by a Bayesian Change-Point Method with ChIP-seq Data (plos.org)	2012	30
**BCP(Histone)**	Genome-Wide Localization of Protein-DNA Binding and Histone Modification by a Bayesian Change-Point Method with ChIP-seq Data (plos.org)	2012	30
**peakC**	peakC: a flexible, non-parametric peak calling package for 4C and Capture-C data	2018	28
**F-seq2**	F-Seq2: improving the feature density based peak caller with dynamic statistics	2021	27
**PolyaPeak**	PolyaPeak: Detecting Transcription Factor Binding Sites from ChIP-seq Using Peak Shape Information	2014	8
**GenoGAM**	GenoGAM: genome-wide generalized additive models for ChIP-Seq analysis	2017	4
**SigSeeker**	SigSeeker: a peak-calling ensemble approach for constructing epigenetic signatures	2017	3
**BinQuasi**	BinQuasi: a peak detection method for ChIP-sequencing data with biological replicates	2018	2
**Ritornello**	Ritornello: high fidelity control-free chromatin immunoprecipitation peak calling	2017	2
**WACS**	WACS: improving ChIP-seq peak calling by optimally weighting controls	2021	1

**Sierra Platinum Service**	The Sierra Platinum Service for generating peak-calls for replicated ChIP-seq experiments.	2018	1
**ChIP-BIT2**	ChIP-BIT2: a software tool to detect weak binding events using a Bayesian integration approach	2021	0
**GoPeaks**	GoPeaks: histone modification peak calling for CUT&Tag	2022	0

Table S2. Applicability of candidate algorithms to test datasets.

Test Dataset	MACS2	HOMER	SICER	PeakRanger	GEM	GoPeaks	SEACR
GSE107690	√	√	√	√	√	×	×
GSE145090	√	√	√	√	√	×	×
GSE133379	√	√	√	√	√	√	×
GSE178668ChIP-seq	√	√	√	√	√	√	√
GSE178668CUT&Tag	√	√	√	√	√	√	√
GSE221437	√	√	√	√	√	√	√

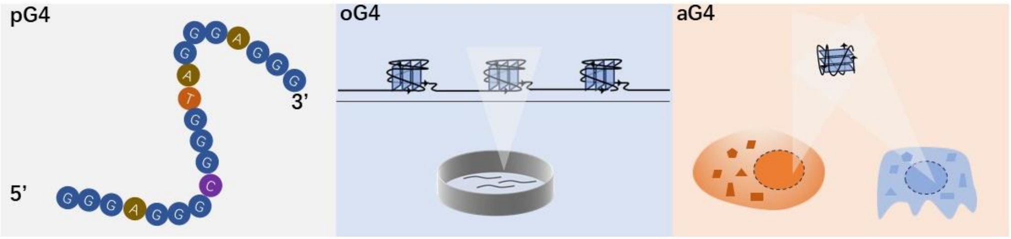

Figure S1. Known G4 information. From left to right: pG4 (putative G4 sequence), oG4 (observed G4), and aG4 (active G4) .

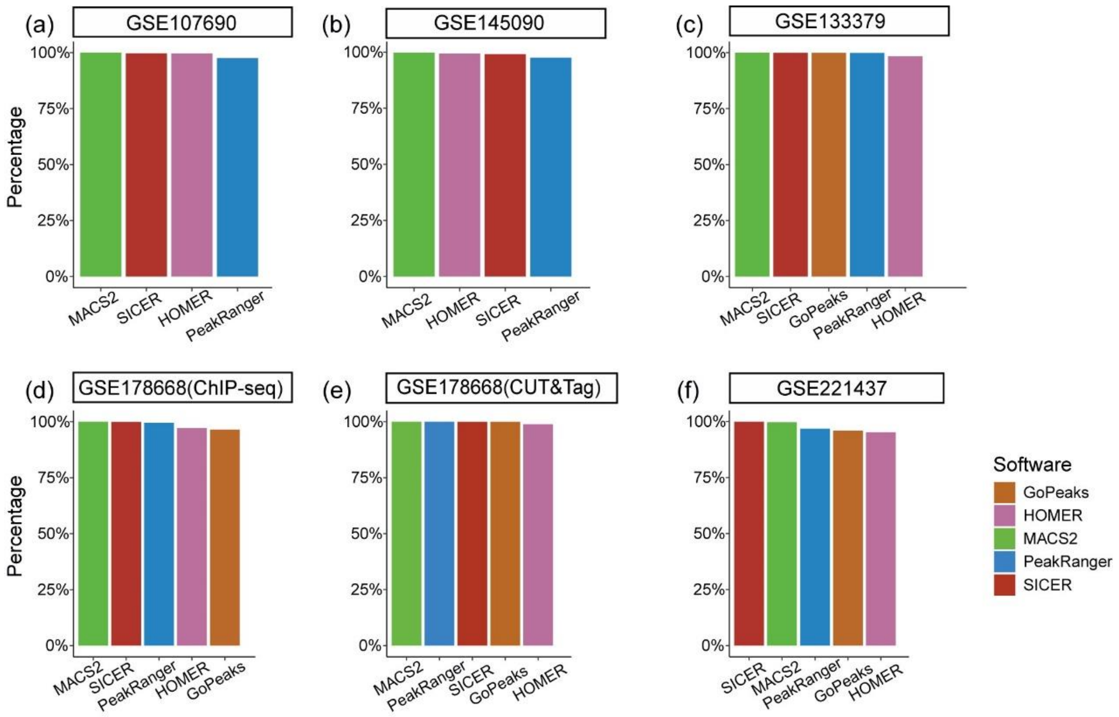

Figure S2. Effectiveness of different strategies in identifying intracellular G4 peaks. The x-axis shows the algorithms, and the y-axis represents the

percentage of overlapping benchmark G4 sites in the candidate peaks identified by each algorithm at the lowest significance threshold. (a) and (b) use single-end test datasets, applicable to four algorithms to be run, while (c)–(f) employ test datasets that accommodate all five algorithms.

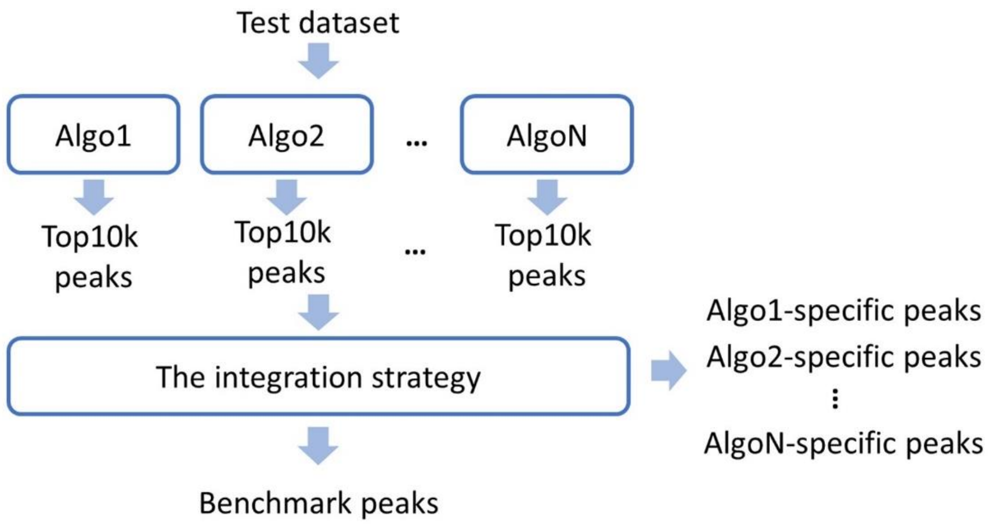

Figure S3. The workflow for generating benchmark peaks and algorithm-specific peaks.
